# Synthesis and antibacterial properties of nanosilver-modified cellulose triacetate membranes for seawater desalination

**DOI:** 10.3762/bjnano.16.100

**Published:** 2025-08-19

**Authors:** Lei Wang, Shizhe Li, Kexin Xu, Wenjun Li, Ying Li, Gang Liu

**Affiliations:** 1 Institute of Chemical and Industrial Bioengineering, Jilin Engineering Normal University, Changchun 130052, Jilin, People’s Republic of China; 2 Jilin Science and Technology Innovation Center of Green Synthesis and New Materials Research and Development, Jilin Engineering Normal University, Changchun 130052, Jilin, People’s Republic of China; 3 School of Biological and food Engineering, Jilin Engineering Normal University, Changchun, 130052, Jilin, People’s Republic of China

**Keywords:** antibacterial properties, cellulose triacetate, nanosilver, seawater desalination

## Abstract

To address the issue of biological pollution in cellulose triacetate (CTA) membranes during seawater desalination, silver (Ag) nanoparticles were incorporated onto the CTA surface using polydopamine (PDA). PDA, which contains phenolic and amino groups, exhibits excellent adhesiveness and provides active sites for the attachment and reduction for Ag nanoparticles. Various characterizations confirm the successful introduction of Ag nanoparticles onto the surface of the PDA-modified CTA (PCTA) membrane and the preservation of CTA microstructures. Antibacterial testing demonstrates that the Ag@PCTA membrane exhibited excellent antibacterial properties. Antibacterial ring experiments revealed significant bactericidal activity against five different bacterial strains, namely, *Bacillus cereus*, *Bacillus thuringiensis*, *Lysinibacillus xylanilyticus*, *Lysinibacillus lparviboronicapiens* and *Burkholderia ambifaria*. Moreover, water flux and salt rejection rates of the Ag@PCTA membrane were comparable to those of the parent CTA membrane.

## Introduction

As the global population continues to grow at a rapid pace, the demand for freshwater resources is escalating. Concurrently, the consumption of freshwater in industrial and agricultural sectors is also witnessing a significant increase [1–2]. On Earth, while oceans cover approximately 75% of the surface, freshwater constitutes merely 2.5% of the total water volume [3–5]. Moreover, 70% of these freshwater resources are located in glaciers, the atmosphere, and soil; rendering them difficult to access and utilize [5–7]. To tackle the challenge of clean water scarcity, desalination of seawater or brackish water have emerged as effective solutions [1,8–9]. According to the literature, desalination technologies can be broadly categorized into two groups, that is, thermal-ased technologies (e.g., multistage flash distillation (MSF) and multieffect distillation (MED)) and membrane-based technologies (e.g., forward osmosis (FO), reverse osmosis (RO), electrodialysis (ED), and nanofiltration (NF)) [10]. Among these, membrane-based desalination stands out due to its numerous advantages, such as low energy consumption, compact footprint, and ease of operation [11–12]. Over the past decade, there has been a substantial increase in research publications related to membrane desalination, with the number reaching 21,233 as of November 2024 (keyword: “membrane desalination”, Web of Science). This surge in research is closely tied to advancements in desalination processes and the introduction of innovative membrane materials [1]. However, the cost of these advanced materials remains higher than that of conventional water supply methods [13]. Additionally, traditional membranes face challenges such as the trade-off between permeability and selectivity, as well as susceptibility to biological pollution [14–15]. Biofouling, which involves attachment, growth, reproduction, and proliferation of microorganisms on the membrane surface, significantly reduces the efficiency of water production [16]. Therefore, there is an urgent need to enhance membrane properties to achieve both high permeability and high selectivity while improving resistance to biological contamination.

Antibacterial nanomaterials have garnered significant attention for their ability to enhance the antibacterial properties of membrane materials, effectively addressing critical issues such as biofouling and microbial contamination in water treatment systems. These nanomaterials, characterized by their high surface area-to-volume ratio, tunable surface properties, and quantum effects, collectively enhance their antimicrobial efficacy [17]. The high surface area-to-volume ratio of nanomaterials facilitates efficient interactions with microorganisms, thereby enhancing their antimicrobial efficacy [18]. Additionally, the surface properties of nanomaterials can be engineered to optimize their antimicrobial activity, making them versatile tools in the development of advanced membrane technologies. Quantum effects, such as localized surface plasmon resonance in metallic nanoparticles, can further enhance antimicrobial properties by generating reactive oxygen species (ROS) that damage bacterial cell membranes and DNA [19]. The incorporation of these nanomaterials into seawater desalination membranes not only significantly improves salt rejection and water flux but also markedly enhances antibacterial properties, thereby extending the operational lifespan of the membranes. The widely used antimicrobial nanoparticles can be categorized based on their composition and properties as follows: metallic nanoparticles, carbon-based nanomaterials, oxide nanoparticles, and composite nanomaterials. Zheng’s group used an in situ synthesis method to embed AgNPs into polyacrylonitrile (PAN) nanofibers, the fabricated Ag/PAN-TFN FO membrane demonstrated excellent antibacterial activity against *Escherichia coli* and *Staphylococcus aureus* with improved water flux and salt rejection. The in situ synthesis method ensures a uniform distribution of AgNPs within the PAN nanofibers, enhancing the hydrophilicity and antibacterial properties of the membrane [20]. Elimelech’s group introduced copper nanoparticles (Cu NPs) onto the surface of thin-film composite (TFC) polyamide RO membranes through a method involving electrostatic functionalization. The functionalized membrane exhibited significant antibacterial activity, reducing the number of attached live bacteria by 80−95% for three different model bacterial strains (*Escherichia coli*, *Pseudomonas aeruginosa*, and *Staphylococcus aureus*). The Cu NPs could be reloaded onto the membrane surface after depletion, indicating the potential for repeated use and long-term application [21]. Zhao and Park’s group incorporated in situ synthesized silver-loaded graphene oxide (GO-Ag) nanoparticles into polyvinyl alcohol/chitosan (PVA/CS) electrospun nanofiber membranes to boost desalination performance. The PVA/CS/GO-Ag membranes exhibited outstanding antibacterial performance against *Escherichia coli* and *Staphylococcus aureus* [22]. Mayyahi introduced TiO_2_ nanoparticles into the polyamide layer of traditional TFC membranes by interfacial polymerization with *m*-phenylenediamine to create a thin film nanocomposite (TFN) membrane for water desalination. The TiO_2_-modified membrane demonstrated significant antibacterial activity against *Escherichia coli.* Under UV light, the survival ratio of bacterial cells reduced to 5% within 4 h; the membrane was completely sterilized within 5 h. This effect is attributed to the photocatalytic properties of TiO_2_, which generate ROS that degrade bacterial membranes [23]. Yang and Wang’s group introduced carbon nanotubes (CNTs) into a mixed cellulose esters (MCE) membrane to create a robust porous bi-layered photothermal membrane (CNT@PEI/MCE) for efficient solar-driven interfacial water evaporation. The CNTs were functionalized with polyethylene imine (PEI) to enhance their dispersibility and to impart antibacterial properties to the membrane. The CNT@PEI/MCE membrane exhibited significant antibacterial activity against *Escherichia coli* [24]. In summary, the in situ reduction method is particularly effective in achieving a uniform distribution of nanoparticles. Among all antibacterial nanomaterials, Ag stands out for its broad-spectrum antimicrobial efficacy, making it a cornerstone in the field of antimicrobial research despite its long-standing history of study. The forms of Ag utilized for antibacterial purposes are diverse and include metallic silver, silver salts, and composites of Ag with other materials. The integration of nanotechnology with Ag, particularly when incorporated into polymer membranes, has demonstrated significant potential for enhancing membrane performance.

CTA fibers boast exceptional hydrophilicity, facile film-forming ability, high salt retention rate, cost effectiveness, environmental friendliness, and resistance to chlorine; making it a promising material for water desalination [25–27]. However, CTA is susceptible to erosion and degradation by waterborne microorganisms because six-membered cyclic ethers in its molecular structure have β-A chains of dehydrated glucose units linked by C–O–C bonds [28]. Therefore, enhancing the antibacterial properties of CTAs is crucial from both academic and practical perspectives.

In this study, we employed the PDA which contains catechol and nitrogen-containing groups, as both a reducing and crosslinking agent to convert Ag ammonia complexes into Ag nanoparticles. These nanoparticles were then grafted onto the surface of a CTA membrane. The entire modification process was conducted under alkaline conditions in an ammonia solution. When compared to the unmodified CTA membrane, the modified membrane exhibited comparable desalination performance. Specifically, for 1.5 g·L^−1^ brackish water under 6 MPa pressure, the modified membrane achieved a water flux of approximately 21.06 L·m^−2^·h^−1^ and a retention rate of around 70%. Additionally, the modified membrane demonstrated enhanced antibacterial properties. This modification process holds significant promise for advancing membrane utilization in industrial settings and facilitating the dissemination of desalination methods for coastal households.

## Results and Discussion

### Structure of membrane material

**Figure 1:**
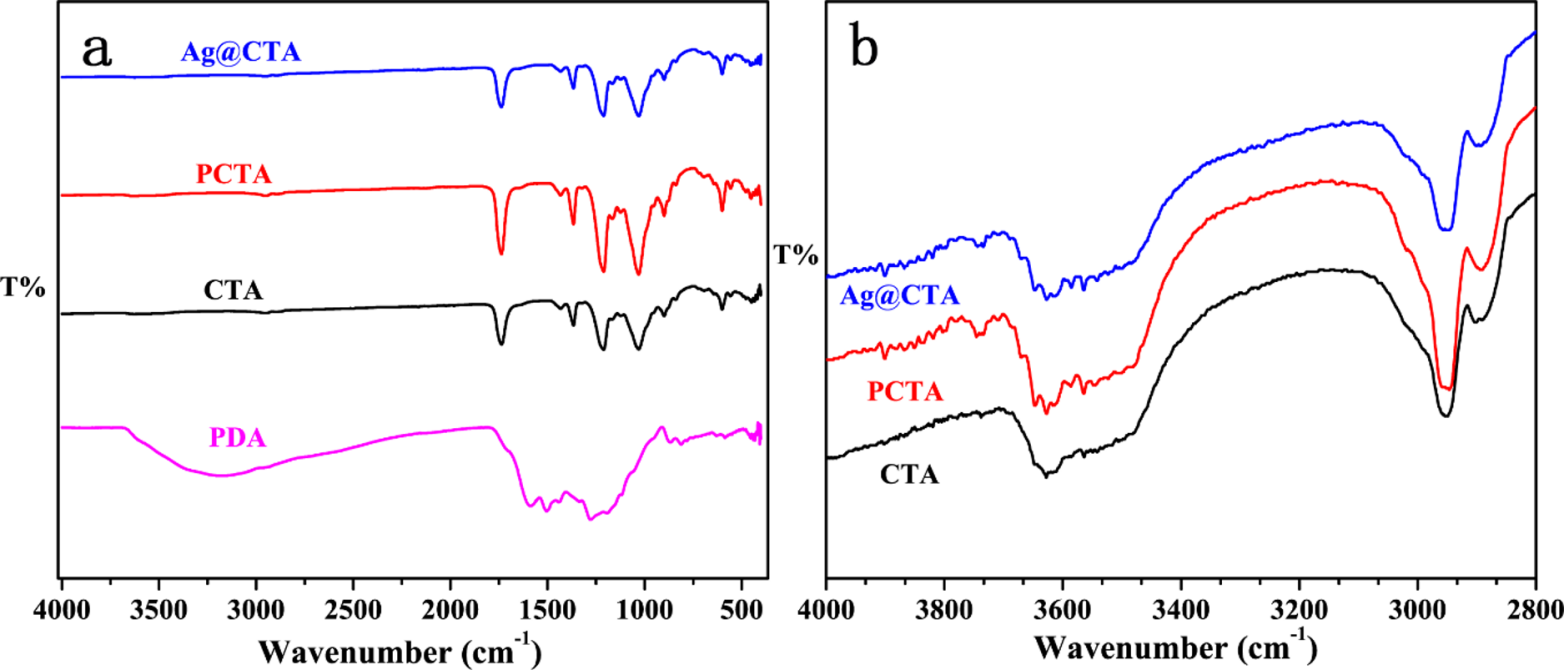
(a) FTIR spectra of PDA, CTA, PCTA, and Ag@PCTA and (b) enlarged view of the FTIR region from 4000 to 2800 cm^−1^ for CTA, PCTA, and Ag@PCTA.

**Figure 2:**
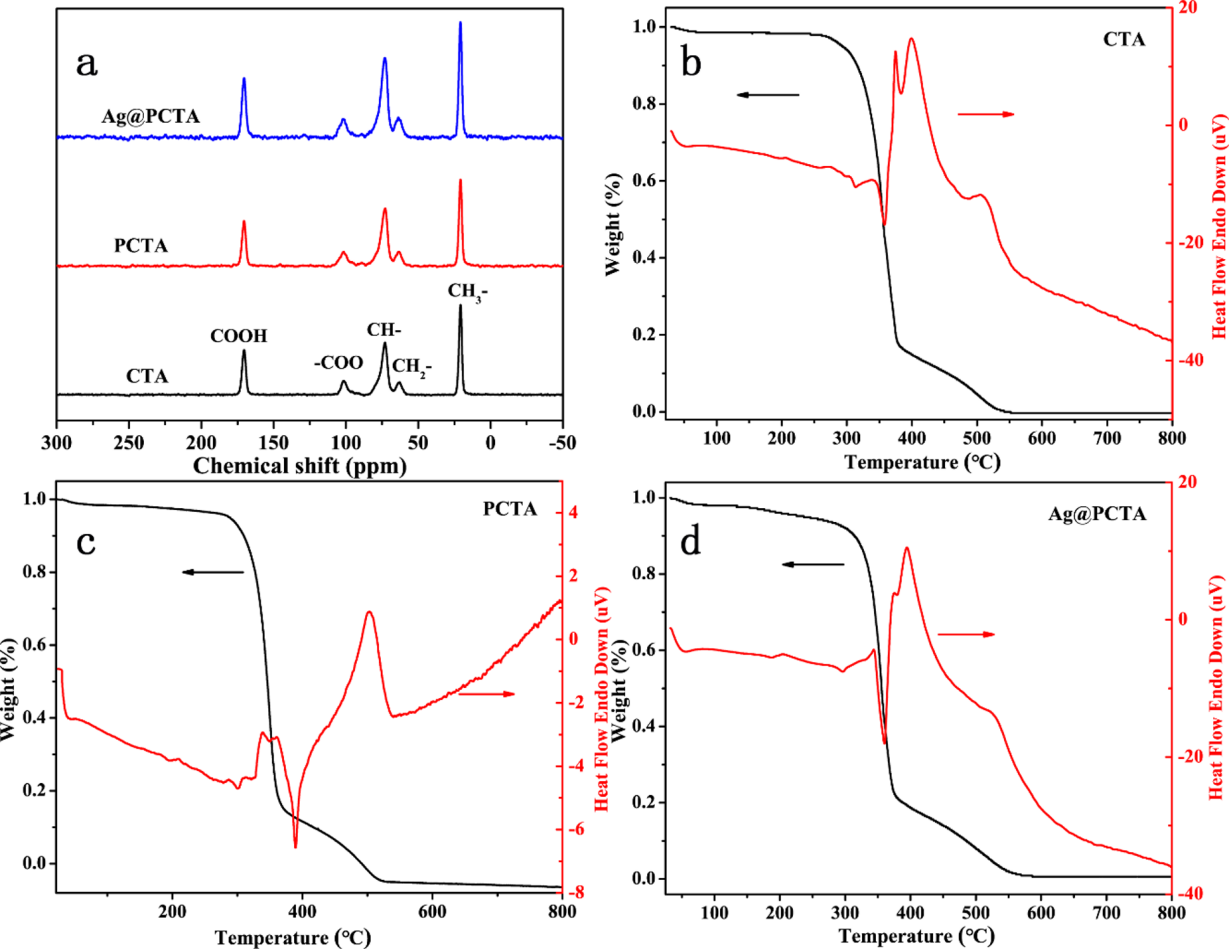
(a) ^13^C NMR spectra and (b–d) TGA and DTA curves of CTA, PCTA, and Ag@PCTA.

To verify the target structure of the synthesized material, Fourier-transform infrared spectroscopy (FTIR) and nuclear magnetic resonance spectroscopy (NMR) were conducted, with the spectra depicted in Figure 1 and Figure 2, respectively. The FTIR bands observed at 3490, 2940, and 1038 cm^−1^ correspond to the stretching vibrations of –OH, C–H, and C–O–C in the CTA membrane. The bands at 1736, 1367, and 1216 cm^−1^ are attributed to C=O, –CH_3_, and C–O in the acetyl groups, respectively. Coating of the CTA surface with PDA does not yield any new characteristic absorption bands in the FTIR spectrum of PCTA and Ag@PCTA. This is because the functional groups in the PDA structure including –OH, –NH_2_, and C=O, which have band positions at 3200–3600 cm^−1^, 3300–3500 cm^−1^, and 1660–1700 cm^−1^, respectively, coincided with those of CTA. Moreover, the modifying agents (PDA and Ag) are present in extremely low quantities relative to the CTA matrix. (For specific details on the content of the modifying layers, please refer to Supporting Information File 1.) The sensitivity of FTIR spectroscopy depends on the concentration of the absorbed species and the path length of the sample. The low concentration of PDA and Ag may result in characteristic peaks being masked by noise; thus no significant differences are observed in the FTIR curves of the three materials. To confirm the successful polymerization of dopamine into PDA, the dark brown layer adhering to the PCTA membrane and the container walls was stripped off and tested via FTIR. The spectrum exhibited characteristic peaks at approximately 1506 cm^−1^ and 1600 cm^−1^, corresponding to the stretching vibrations of the indole ring structure (Figure 1) [29]. This result indicates that dopamine was successfully polymerized on the surface of the CTA membrane under the given conditions. The low concentration of Ag nanoparticles in Ag@PCTA also results in minimal spectral changes compared to PCTA. The Ag nanoparticles are present in trace amounts, which may not significantly alter the overall FTIR spectrum.

The internal structures of the three membranes remain unchanged, as confirmed by ^13^C solid-state NMR spectroscopy (Figure 2a). The chemical shift at 172 ppm is attributed to the sp^2^-hybridized carbon of the carbonyl group in the acetyl group (–COOCH_3_), while the chemical shifts at 20.3, 62.9, and 72.4 ppm correspond to the sp^3^-hybridized carbons of methyl and methylene groups. The chemical shift for carbon atoms adjacent to two oxygen atoms is located at 101.4 ppm. Similar to the FTIR results, the NMR spectra show minimal differences among the three samples, with no distinct peaks attributable to PDA. This is primarily due to the particularly low content of PDA in the PCTA composite membrane, which significantly impacts the detection of PDA in NMR. The low concentration of PDA results in signal intensities that are much lower compared to the dominant signals from other components in the sample, such as CTA. Although modern NMR instruments have improved sensitivity, there is still a practical detection threshold. If the concentration of PDA falls below this threshold, the signal may not be detectable or may be too weak to provide meaningful information. These factors make it challenging to detect and quantify PDA peaks in the NMR spectra, resulting in the absence of observable characteristic peaks of PDA.

Thermogravimetric analysis (TGA) was used to evaluate the membrane’s thermal stability, with the results presented in Figure 2b–d. The minimal differences observed among the three TGA curves also indicate the low content of the modifying agents (PDA and Ag) relative to the CTA matrix. This low concentration results in negligible changes in the overall thermal behavior of the composite materials, as detected by TGA. This observation also suggests that the interaction between Ag and CTA or between PDA and CTA did not significantly alter the decomposition pathways of the CTA, consistent with the FTIR and NMR data (Figure 1 and Figure 2). The three curves exhibit almost identical weight loss patterns, that is, an initial weight loss of about 5% below 300 °C, attributed to volatile substances or absorbed water; a noticeable weight loss in the temperature range of 300–370 °C, due to the thermal degradation of CTA; and slow weight loss after 370 °C, caused by the combustion of degraded products.

Differential thermal analysis (DTA) reveals distinct transition temperatures associated with endothermic and exothermic events for the three materials (Figure 2b–d). These differences in transition temperatures confirm the successful surface modification of CTA with PDA and the subsequent successful deposition of Ag nanoparticles. The unique thermal signatures observed in the DTA curves provide clear evidence of the successful sequential functionalization of the CTA membrane. In addition, at a temperature of 800 °C, both the CTA and PCTA films exhibit a residual mass of 0%, indicating complete decomposition or volatilization, leaving no significant inorganic residues. In contrast, the Ag@PCTA film shows a residual mass of 0.006% at 800 °C. This small but detectable residual mass can be attributed to the presence of Ag nanoparticles within the film. Given the extremely low mass ratio of Ag in the Ag@PCTA film (0.0054 mg/mg), this residual mass is consistent with the expected contribution from the Ag nanoparticles. The stability of Ag, even in such low quantities, is indicated by their resistance to decomposition or volatilization at temperatures up to 800 °C. However, due to the extremely low content of Ag, the residual mass is within the detection limit of the TGA instrument, and thus the result cannot exclude the possibility of instrumental error.

**Figure 3:**
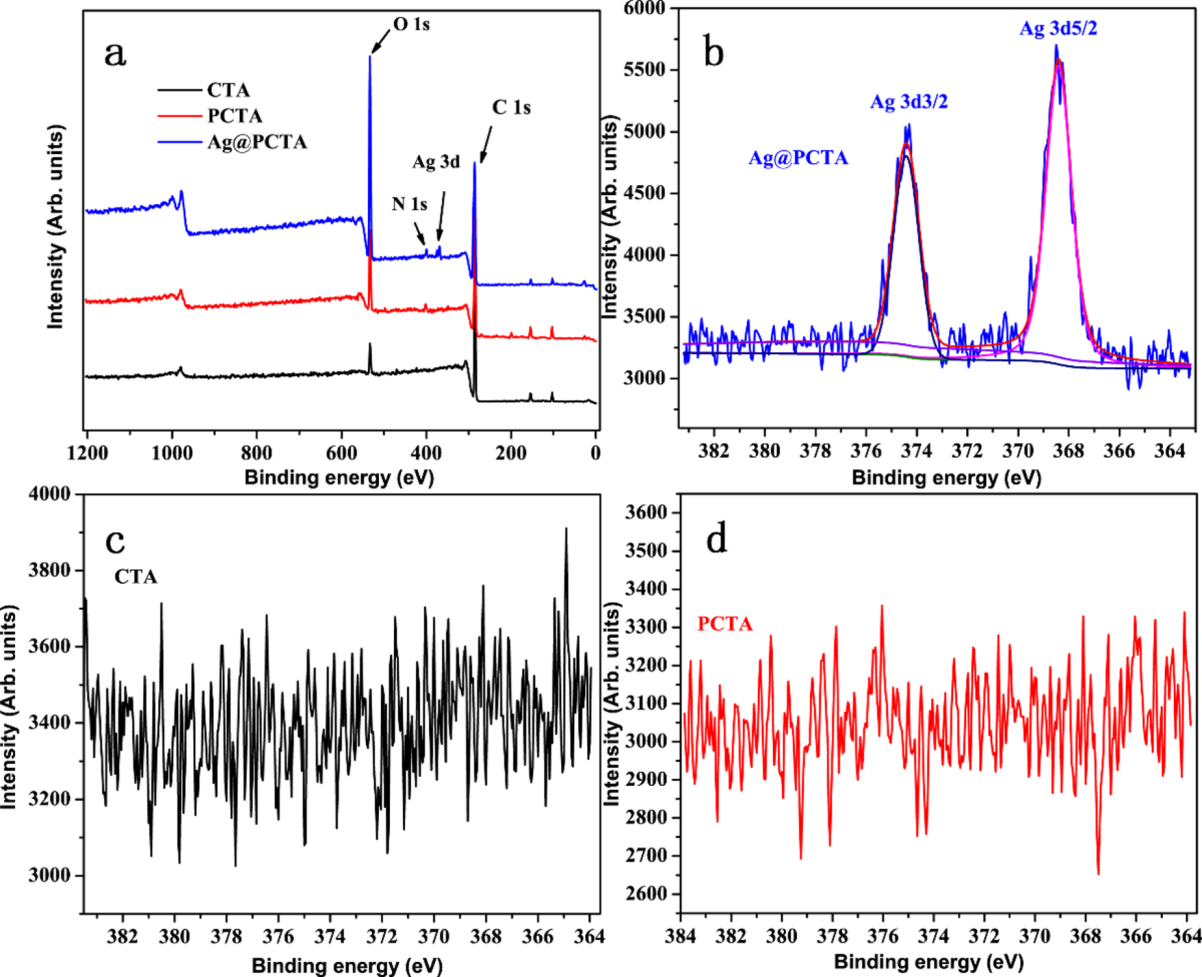
(a) XPS wide scan spectrum of CTA, PCTA, and Ag@PCTA. High-resolution XPS spectra of Ag in (b) CTA, (c) PCTA, and (d) Ag@PCTA.

X-ray photoelectron spectroscopy (XPS) is utilized to analyze the surface chemical compositions of Ag@PCTA, PCTA, and CTA. Figure 3a shows the XPS wide scan spectrum of CTA, PCTA, and Ag@PCTA. The C 1s peak at a binding energy (BE) of 284.94 eV and the O 1s peak at a BE of 532.44 eV can be clearly observed in the spectrum of CTA. The surface C/O molar ratio of the CTA is about 12.2, indicating that CTA is composed of C, O, and H; the carbon content in CTA is higher than the oxygen. In the wide-scan spectrum of the PCTA film, the presence of the PDA layer on the CTA surface can be deduced from the appearance of the new peak component at a BE of about 400.02 eV associated with N from PDA. The surface C/O molar ratio of PCTA was calculated to be 4.04, which is close to the theoretical value of C/O in PDA (4.0). The C/O ratio of PCTA is close to the theoretical value of PDA, which proves the successful modification of PDA on the surface of CTA. At the same time, it indicates that the thickness of PDA layer exceeds the maximum thickness detectable by XPS which has a detectable thickness of organic matrix of about 7.5 nm [30]. In the wide-scan spectrum of the Ag@PCTA film, a new peak at a BE of 368.42 eV was observed, corresponding to Ag 3d. This peak originates from the silver species in Ag(NH_3_)_2_OH, confirming the presence of silver on the film surface. The C/O molar ratio of Ag@PCTA was calculated to be 2.06, which is lower than the C/O ratio of the PCTA film. This decrease in the C/O ratio is likely attributed to hydrogen bonding interactions between the –OH groups in Ag(NH_3_)_2_OH and the PDA layer. These interactions enhance the oxygen content relative to carbon, thereby reducing the C/O ratio. Collectively, these results provide compelling evidence for the successful surface modification of CTA with PDA and the subsequent immobilization of Ag on the PCTA surface via coordination bonds. In order to clarify the valence state of Ag, XPS was employed to provide detailed information about the elemental composition and chemical states of the Ag@PCTA membrane. In the high-resolution Ag 3d spectrum of Figure 3b, the characteristic BEs of Ag 3d_5/2_ and Ag 3d_3/2_ are observed at around 368.5 and 374.3 eV, respectively. These values are indicative of the presence of Ag^0^, which was also well established in the literature [29–34]. In comparison, no peaks of Ag are observed in CTA and PCTA (Figure 3c,d).

Under alkaline conditions (pH > 7.5), dopamine can spontaneously polymerize into PDA in the presence of oxygen (Supporting Information File 1, Figure S1a) [31,35–36]. During the preparation of PCTA, alkaline solution immediately triggered the polymerization of dopamine monomers, accompanied by a color change from light brown to deep brown or black after 24 h, as illustrated in Supporting Information File 1, Figure S2. In this process, the protons generated from the oxidation process are consumed, shifting the equilibrium towards the formation of PDA, which is rich in phenol and amino groups [37]. Additionally, free radical polymerization and physical self-assembly pathways contribute the formation of cross-linked PDA with multiple active sites (e.g. phenol and secondary amino groups) [36,38–39]. The phenol and amino groups in the PDA structure play crucial roles in these processes. Herein, the catechol and nitrogen-containing groups in PDA are exploited to absorb [Ag(NH_3_)_2_]^+^ ions onto the PDA-coated surface. The metal-binding ability and the weak reducibility of PDA are exploited to reduce the absorbed [Ag(NH_3_)_2_]^+^ ions into Ag nanoparticles, which are then immobilized onto CTA membrane (Supporting Information File 1, Figure S1b,c) [40–43]. This process bypasses the need for conventional environmentally unfriendly oxidation and activation processes, as reported in the literature [44–46]. Upon the application of Ag[NH_3_]_2_OH to the surface, the membrane underwent a slight color change, acquiring a metallic luster (Supporting Information File 1, Figure S3). This change is likely attributed to the presence of Ag nanoparticles. Ag nanoparticles are known for their unique optical properties, which can manifest as a metallic sheen when they are present on a substrate [47].

**Figure 4:**
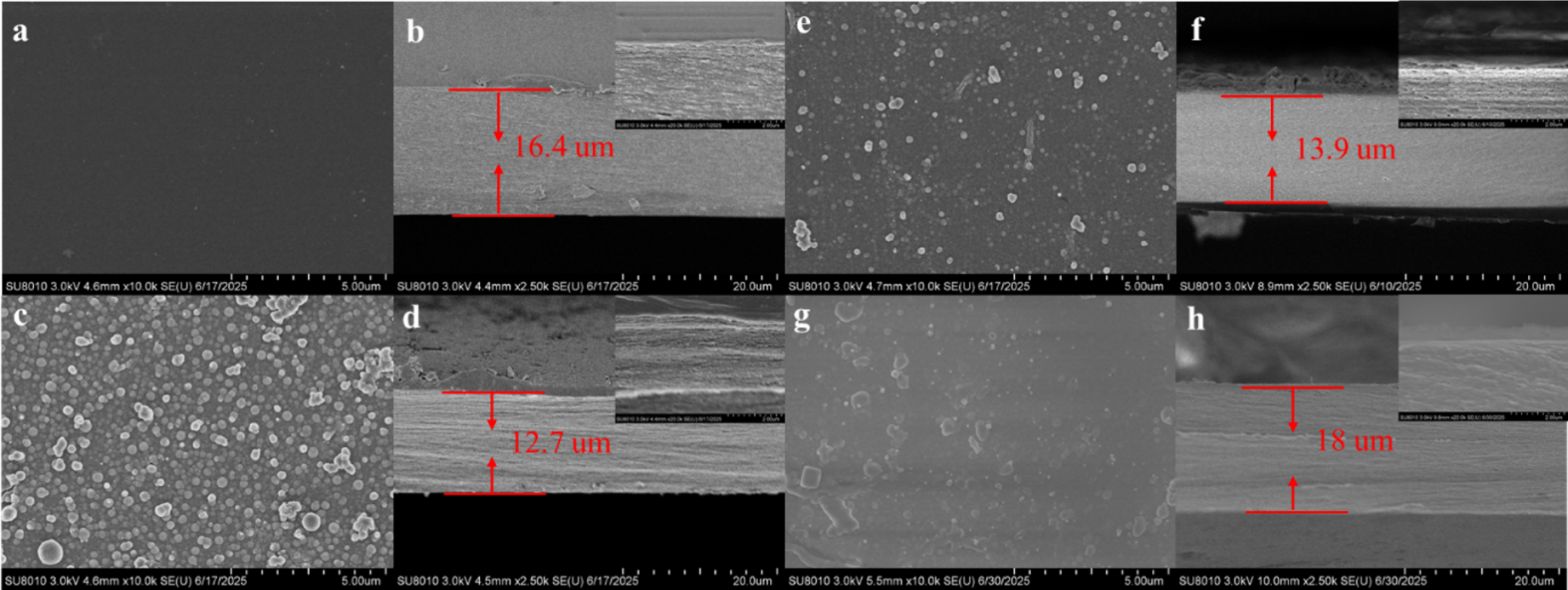
(a, c, e) Top-view and (b, d, f) cross-sectional SEM images of (a, c) CTA, (c, d) PCTA, and (e, f) Ag@PCTA membrane surfaces. The inset (top right) provides a higher magnification view of the selected region, highlighting the detailed arrangement of PDA and Ag nanoparticles on the membrane surface. Morphological changes in (g) surface (g) and (h) cross section of Ag@PCTA after desalination, with an enlarged view of the cross-sectional layer in the upper right corner.

Surface alterations of the membranes are documented using scanning electron microscopy (SEM) (Figure 4). The CTA membrane’s surface appears smooth and uniform, devoid of visible defects (Figure 4a). Cross-sectional SEM images, along with high-magnification views, reveal a dense, compact structure indicative of high polymer packing density (Figure 4b). The application of the PDA coating results in uniformly sized, densely packed spheres that adhere uniformly to the CTA membrane’s surface, providing active sites for further functionalization (Figure 4c). This PDA layer imparts functional groups, such as catechol and amino groups, which facilitate the subsequent reduction and immobilization of Ag nanoparticles. Upon Ag deposition, the PCTA membrane’s surface exhibits evenly distributed Ag nanoparticles nestled within the PDA layer (Figure 4e). These Ag nanoparticles, typically appearing as discrete entities, are interspersed among the PDA microspheres, contributing to the membrane’s antimicrobial properties essential for mitigating biofouling in desalination applications. The cross-sectional SEM images, along with high-magnification views of the PCTA membrane (Figure 4d), reveal a dense and compact internal structure. At higher magnifications, a thin PDA layer is clearly visible on the surface of the CTA membrane, indicating successful modification of the CTA membrane with PDA. The cross-sectional SEM images of the Ag@PCTA membrane illustrate a well-defined PDA layer that maintains its uniformity and adhesion to the underlying CTA substrate (Figure 4f). Notably, Ag nanoparticles are not discernible in the cross-sectional view, indicating their confinement within the PDA layer. The underlying CTA layer remains unchanged, displaying no discernible differences between Ag@PCTA and PCTA membranes.

Additionally, to further support our findings and provide a more comprehensive understanding of the elemental distribution, we have included EDS elemental mapping for the images in Figure 4b,d,f in Supporting Information File 1, Figure S4. The EDS maps confirm the presence of N and Ag within the layers of PCTA and Ag@PCTA, respectively, and provide additional evidence for the structural and compositional analysis. The EDS mapping confirms that both PDA and Ag have been successfully deposited onto the surface of the CTA membrane, thereby corroborating the conclusions drawn from the SEM images.

To investigate the morphological changes of Ag@PCTA during the desalination process, SEM was used to examine the surface morphology of the coating and to identify any signs of degradation or loss. The surface images reveal delamination, partial loss of PDA microspheres, and Ag nanoparticles, as well as the presence NaCl within the coating (Figure 4g). These observations are attributed to the prolonged exposure to saline water, which causes chemical degradation of the PDA layer. Also, the high ionic strength and potential presence of oxidizing agents in brackish water can lead to the breakdown of the PDA matrix. The cross-sectional SEM image shows that the PDA layer remains present on the surface of the Ag@PCTA membrane, with a relatively dense structure (Figure 4h). High-magnification views clearly depict the PDA layer. EDS is again used to analyze the elemental composition of the coating. It can be seen that the coating has been compromised and Na^+^ has penetrated the coating. Furthermore, the AgNPs partially aggregate or migrate within the PDA layer due to changes in the local chemical environment (Supporting Information File 1, Figure S5).

### Desalination performance of membranes

**Figure 5:**
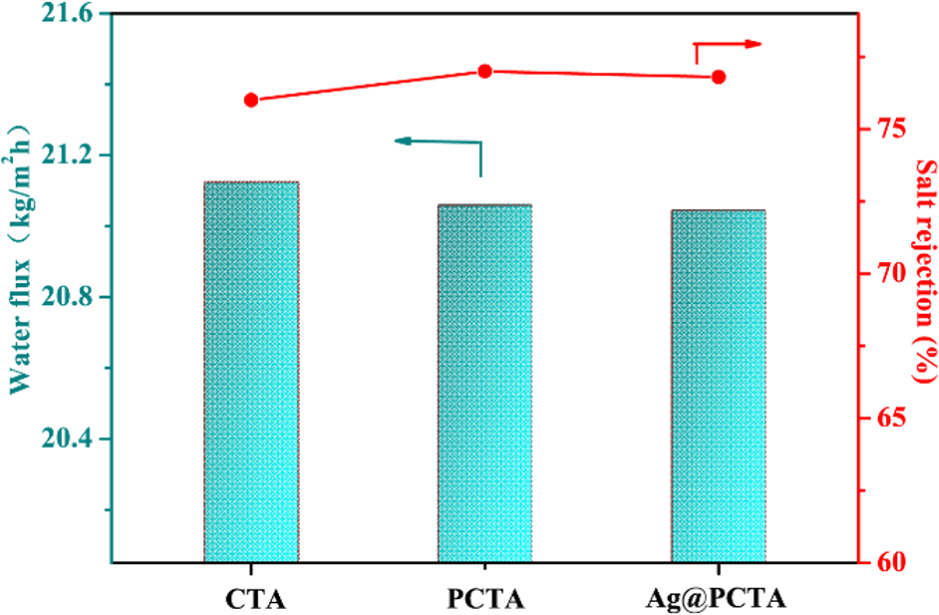
Water flux and salt rejection rate (NaCl) for the membranes of CTA, PCTA and Ag@PCTA.

In the RO process, the water flux and salt rejection rate of the membranes were evaluated before and after modification. At a pressure of 6 MPa, the water fluxes of Ag@PCTA, PCTA, and CTA were measured at 21.05, 21.06, and 21.13 L·m^−2^·h^−1^, respectively, while the salt rejection rates were 76.8%, 77.1%, and 76.3%, respectively (Figure 5). Comparisons of Ag@PCTA with CTA and PCTA reveal that the water flux and rejection rate are almost indistinguishable among the three membranes. This minimal variation in performance can be attributed to two primary factors. First, the abundant –OH and amino groups in PDA promote hydrogen bonding with water molecules, thereby enhancing the hydrophilicity of the membrane surface. This effect is further amplified by the dopamine coating, which not only increases the membrane thickness but also augments its hydrophilicity. The increased hydrophilicity facilitates water transport through the membrane, potentially contributing to the observed water flux. Second, the modification process does not alter the intrinsic pore structure of the CTA membrane. As a result, the pathways for water molecules and salt ions remain unchanged, leading to no significant differences in the transport properties of the membranes. The consistent pore size and structure ensure that the water flux and salt rejection rates remain similar across the modified and unmodified membranes. The combined effects of these factors result in minimal observable differences in water flux and rejection rate among the three membranes.

### Antibacterial properties of membranes

**Figure 6:**
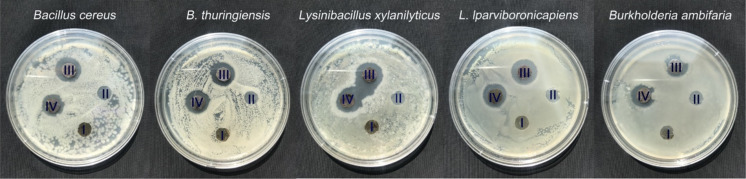
Antibacterial effects of various membranes on different bacterial strains. Inserts I to IV represent PCTA, CTA, and Ag@PCTA membranes with different Ag concentrations (0.05 mol·L^−1^, 0.02 mol·L^−1^), respectively.

The antibacterial effect of the membrane before and after modification is illustrated in Figure 6. CTA, PCTA, and Ag@PCTA exhibit different antibacterial properties against *Bacillus cereus*, *Bacillus thuringiensis*, *Lysinibacillus xylanilyticus*, *Lysinibacillus lparviboronicapiens* and *Burkholderia ambifaria* after 24 h of contact of the membrane with the above bacteria. The modified membrane of Ag@PCTA demonstrates significant disinfection and sterilization effects on all tested strains, evidenced by a pronounced inhibitory ring. Notably, bacteria surrounding the Ag@PCTA membrane are unable to grow, indicating the membrane’s potent antibacterial activity. The antibacterial activity of Ag ions was attributed to their interactions with S-, O-, and N-containing groups in the bacteria [48]. Dai and Bruening have reported that Ag nanoparticle-containing films have the same antibacterial effect as films containing Ag ions [49]. The nanoparticle-containing films may be much better because they minimize the amount of Ag ions absorbed in the body. The present work confirms the good antibacterial activity of Ag nanoparticles on the surface of the Ag@PCTA film. It is evident despite a decrease in nano Ag dosage from 0.05 mol·L^−1^ to 0.02 mol·L^−1^ (from III to IV in Figure 6), the size of the inhibitory ring remains almost unchanged, highlighting the robust antibacterial performance of the Ag@PCTA membrane even at lower Ag concentrations. In contrast, the CTA and PCTA membranes show no inhibitory effect on the growth of the tested strains, underscoring the significant enhancement in antibacterial properties achieved through Ag NPs modification.

To provide a comprehensive understanding of the antibacterial properties of our material, we conducted a detailed comparison with existing literature on the use of Ag to enhance the antibacterial properties of desalination membranes. This comparison encompasses the types of bacteria inhibited and the various forms in which Ag exists, including AgNPs, Ag^+^ ions, and other Ag-based compounds. The detailed comparative data are systematically presented in Table 1. From Table 1 we can find, our material demonstrates antibacterial efficacy that is comparable to other Ag-enhanced desalination membranes reported in the literature. This indicates that the incorporation of Ag in our material is effective in inhibiting bacterial growth and biofouling. Notably, the spectrum of bacteria against which our material is effective has not been previously documented in other studies. This finding expands the knowledge regarding the antibacterial activity associated with silver nanoparticles.

**Table 1:** Types of Ag and their antibacterial properties in desalination membranes as reported in the literature.

Morphology of silver	Bacterial strain	Antibacterial efficacy	Ref.

AC/Ag-n	*E. coli; S. enteritidis*	100% *E. coli;* 98% *S. enteritidis*	[50]
Tf-TA2/Ag	*S. aureus; E. coli*	99.99% *E. coli;* 99.99% *S. aureus*	[51]
Ag-PDA@wood	*E. coli*	*E. coli*	[52]
GO@PAMAM@Ag	*E. coli; S. aureus*	*E. coli; S. aureus;*	[53]
GO/Ag nanocomposite	*E. coli*	over 95% *E. coli*	[54]
Ag/PPy	*E. coli*	*E. coli*	[55]
Ag NPs	*E. coli; Corynebakterium glutamicum*	*E. coli; Corynebakterium glutamicum*	[56]
Ag/AgCl NPs	*E. coli*; *S. aureus*; *B. subtilis*	99.87% *E. coli;* 99.78% *S. aureus*; 99.96% *B. subtilis*	[57]
GOQD/AP	*E. coli*	99.9% *E. coli*	[58]
Ag/PVDF	*E. coli*	99% *E. coli*	[59]
PAN-Ag	*B. subtilis; E. coil*	*B. subtilis; E. coil*	[60]
TA-Fe-PEI/Ag	*E. coli; B. subtilis*	100% *E. coli; 100% B. subtilis*	[61]
GO-Ag pH_x_	*E. coli*	99.1–99.9% *E. coli*	[62]
CF-Ag-PPy	*E. coli*; *S. aureus*	99.95% *E. coli*; 99.95% *S. aureus*	[63]
Ag/TNTs	*E. coli*; *S. epidermidis*	99.8% *E. coli*; 99.3% *S. epidermidis*	[64]
AC-Ag	*E. coli; S. aureus*	99% *E. coli;* 99% *S. aureus*	[65]
AgNPs-PDA hydrogel	*E. coli*	*E. coli*	[66]

## Conclusion

To address the issue of microbial contamination in desalination membranes, we have developed an antibacterial Ag@PCTA membrane. Utilizing the catechol and nitrogen-containing groups in PDA as both crosslinking and reducing agents, we successfully introduced antimicrobial Ag NPs onto the surface of the CTA membrane. This modification confers significant antibacterial activity against five bacterial strains (*Bacillus cereus*, *Bacillus thuringiensis*, *Lysinibacillus xylanilyticus*, *Lysinibacillus lparviboronicapiens*, and *Burkholderia ambifaria*) without compromising the membrane’s water flux. The PDA coating enhances the hydrophilicity of the membrane and maintains its water permeation properties. This membrane holds significant potential for enhancing the performance of seawater desalination membranes. As research in this field continues to advance, the potential for further improvements and new applications of nanomaterials in membrane technology remains promising.

## Experimental

### Materials

CTA was purchased from DAICEL (China) Investment Co., Ltd. Dopamine hydrochloride (98%), tris(hydroxymethyl)aminomethane hydrochloride (standard buffer substance, 99.9% (titration)), trifluoromethanesulfonic acid (99.5%), and AgNO_3_ (0.1 mol·L^−1^ in H_2_O) were all sourced from Aladdin Chemical Reagent Co., Ltd (Shanghai, China). Ammonia was obtained from Harbin Dongfang Chemical Reagent Co., Ltd. All chemical reagents were utilized as received without further purification.

*Bacillus cereus, Bacillus thuringiensis*, *Lysinibacillus xylanilyticus, Lysinibacillus lparviboronicapiens*, and *Burkholderia ambifaria* were isolated and purified from farmland, Jilin province, China. Peptone, beef extract, sucrose, and NaCl, purchased from Tianjin Sanjiang Chemical Technology Co. Ltd., were employed to prepare the NB culture medium.

### Characterizations

TGA was carried out on a Shimadzu DTG-60/DTG-60A thermogravimetric analyzer. The heating rate was 10 °C·min^−1^, and the atmosphere was air. FTIR measurements were performed using a PerkinElmer spectrometer, utilizing single-reflection ATR technology and a diamond crystal. Bruker AV400 and VARIAN 300 NMR spectrometers were used to collect ^13^C NMR spectra at 298 K. CDCl_3_ solvent was used as internal standard in ^13^C NMR experiment (δ 77.00 ppm). A Scienta ESCA200 apparatus was used to record XPS spectra. The morphology of the membrane was monitored using a field-emission SEM (FE-SEM, JEOS JSM6700F). An InoLab Cond 7310 conductivity meter was used to measure ion concentration.

### Fabrication of CTA membrane

The membranes were prepared according to previous protocols with modifications [67]. Initially, the raw materials for the CTA film were dried in a vacuum oven at 80 °C for 12 h. Subsequently, 1.31 g of dried powder was added to a round bottom flask. A mixed solution of 1,4-dioxane (6.24 g) and acetone (2.49 g) in a mass ratio of 5:2 was added to the flask while stirring vigorously for 12 h until complete dissolution of the materials was achieved. The resulting dissolved viscous solution was then sealed and stored for 24 h to allow for defoaming, resulting in a uniform, transparent, and bubble-free casting solution. The CTA film was prepared using the solution via the scraping method. The resultant membrane was exposed to air for 30 s before being transferred to water for solvent replacement, a process that lasted for 3 days. During the period, the water was changed once a day.

### Preparation of dopamine/hydrochloric acid buffer solution

The buffer solution was prepared by modifying the reported method [35–36]. Typically, 0.121 g of Tris was dissolved in 100 mL of water, followed by gradual addition of dilute hydrochloric acid until the solution reached a pH of 8.5. Subsequently, 10 mg of dopamine hydrochloride (98%) was dissolved in 5 mL of the tris/HCl solution to prepare a 2 mg·mL^−1^ dopamine solution. The CTA membrane surface was then coated with the buffer solution and allowed to dry for 24 h. Under weakly alkaline conditions, the dopamine underwent polymerization reaction, forming a PDA layer on the CTA membrane surface, resulting in the PCTA membrane.

### Preparation of silver ammonia solution

Initially, an appropriate volume of 0.10 mol·L^−1^ AgNO_3_ was taken from a volumetric flask and diluted to either 0.05 or 0.02 mol·L^−1^. Subsequently, ammonia solution was added dropwise to the diluted AgNO_3_ solution, causing the solution to gradually become turbid. As the addition of ammonia solution continued, the turbidity diminished until the solution became clear, indicating the formation of [Ag(NH_3_)_2_]OH.

### Grafting of nano Ag onto PCTA membrane

The PCTA membrane was immersed in an [Ag(NH_3_)_2_]OH solution and underwent static reaction for 8 h. During this process, the phenolic hydroxy groups in PDA facilitated the reduction of Ag(NH_3_)_2_^+^ particles, resulting in the formation of the Ag@PCTA membrane. Finally, the membrane was cleaned three times with ultrapure water and then placed in a vacuum drying oven for further use.

### Characterization of antibacterial performance by disk diffusion method

The modified CTA membrane and unmodified membrane were each cut into approximately 78.5 mm^2^ pieces, which were disinfected with ultraviolet light for 30 min. A lawn of the experimental strain was prepared on NB medium (0.3 wt % beef extract, 0.1 wt % yeast extract, 0.5 wt % peptone, 1% sucrose, and 0.5 wt % NaCl), with an original concentration of 1 × 10^6^ CFU·mL^−1^ through spreading 25 µL bacterial liquid on each plate. The sterilized membranes were then placed on the top of the plate containing the attached bacterial strain and then incubated at 28 °C for 24 h. The antibacterial efficacy of each membrane was assessed by measuring the size of the inhibition zone against different bacterial strains. The strains selected for the experiment were *Bacillus cereus*, *Bacillus thuringiensis*, *Lysinibacillus xylanilyticus*, *Lysinibacillus lparviboronicapiens* and *Burkholderia ambifaria*.

### RO experiment

The reverse osmosis device was designed by our laboratory for conducting seawater desalination experiments. The feed comprising a 1.5 g·L^−1^ NaCl aqueous solution was set with a test pressure of 6 MPa and a temperature of 25 ± 0.5 °C. Prior to commencing the test, the membrane was compacted. During the experimentation, it was imperative to record both the volume of permeate and the corresponding time on the permeate side. The permeation flux of the membrane was calculated utilizing the following formula (1).


[1] $$J_{\text{w}}=\frac{V}{A\times \Delta_{t}}.$$

In the formula, *J*_w_ is the water flux, L·m^−2^·h^−1^; *V* is the volume of the permeate, L; *A* is the effective testing area of the membrane, m^2^; and Δ*_t_* is the duration of sampling, h.

The calculation for the salt rejection rate was as follows:


[2] $$R=\left(1-\frac{C_{1}}{C_{0}}\right)\times 100\%.$$

In the formula, *R* is the retention rate, %; *C*_1_ is the concentration of the solution passing through the permeate side; *C*_0_ is the concentration of the solution on the feed side. The salt concentration in the solution was determined by testing the conductivity of the solution using a conductivity meter.

## Supporting Information

File 1 Additional experimental data.

## Data Availability

All data that supports the findings of this study is available in the published article and/or the supporting information of this article.

## References

[1] Ruan G, Wang M, An Z, Xu G, Ge Y, Zhao H (2021). Membranes.

[2] Mukherjee M, Roy S, Bhowmick K, Majumdar S, Prihatiningtyas I, Van der Bruggen B, Mondal P (2022). Process Saf Environ Prot.

[3] Saboori H (2023). Sustainable Energy Technol Assess.

[4] Salehi A A, Ghannadi-Maragheh M, Torab-Mostaedi M, Torkaman R, Asadollahzadeh M (2021). Sep Purif Rev.

[5] Carpenter S R, Stanley E H, Vander Zanden M J (2011). Annu Rev Environ Resour.

[6] Aranguren-Díaz Y, Galán-Freyle N J, Guerra A, Manares-Romero A, Pacheco-Londoño L C, Romero-Coronado A, Vidal-Figueroa N, Machado-Sierra E (2024). Water.

[7] Sivakumar B (2011). Hydrol Sci J.

[8] Liu H, Ji D, An M, Kandeal A W, Thakur A K, Elkadeem M R, Algazzar A M, Abdelaziz G B, Sharshir S W (2023). Process Saf Environ Prot.

[9] Qin X, Qin X, Xu X, Zhao J, Gui Y, Guo H, Mao J, Wang Y, Zhang Z (2023). Desalination.

[10] Panagopoulos A, Haralambous K-J (2020). Mar Pollut Bull.

[11] Ahmad N A, Goh P S, Yogarathinam L T, Zulhairun A K, Ismail A F (2020). Desalination.

[12] Yusuf A, Sodiq A, Giwa A, Eke J, Pikuda O, De Luca G, Di Salvo J L, Chakraborty S (2020). J Cleaner Prod.

[13] Gude V G (2016). Water Res.

[14] Goh P S, Lau W J, Othman M H D, Ismail A F (2018). Desalination.

[15] Saleem H, Zaidi S J (2020). Desalination.

[16] Rapenne S, Barbe C, Schaule G, Strathmann M, Grobe S, Croué J-P, Mondamert L, Hijnen W, van der Kooij D, Manes C L d O, Drioli E, Criscuoli A, Macedonio F (2011). Development of tools for RO fouling characterization and understanding. Membrane-Based Desalination: An Integrated Approach (MEDINA).

[17] Smith S C, Rodrigues D F (2015). Carbon.

[18] Parvin N, Joo S W, Mandal T K (2025). Antibiotics (Basel, Switz).

[19] Mammari N, Lamouroux E, Boudier A, Duval R E (2022). Microorganisms.

[20] Pan S-F, Ke X-X, Wang T-Y, Liu Q, Zhong L-B, Zheng Y-M (2019). Ind Eng Chem Res.

[21] Ben-Sasson M, Zodrow K R, Genggeng Q, Kang Y, Giannelis E P, Elimelech M (2014). Environ Sci Technol.

[22] Kang Q, Zhai Y, Zhao F, Yang L, Yang Y, Park H-D, Li Z, Chen H, Sun G (2024). Chem Eng Res Des.

[23] Al Mayyahi A (2018). Membranes.

[24] Li Y, Cui X, Zhao M, Xu Y, Chen L, Cao Z, Yang S, Wang Y (2019). J Mater Chem A.

[25] Lee K P, Arnot T C, Mattia D (2011). J Membr Sci.

[26] Li S, Wang X, Guo Y, Hu J, Lin S, Tu Y, Chen L, Ni Y, Huang L (2022). J Cleaner Prod.

[27] Waheed S, Ahmad A, Khan S M, Gul S-e, Jamil T, Islam A, Hussain T (2014). Desalination.

[28] Li F, Fei P, Cheng B, Meng J, Liao L (2019). Carbohydr Polym.

[29] Yang F, Wang S, Li Z, Xu Y, Yang W, Yv C, Yang D, Xie Y, Zhou W (2022). J Colloid Interface Sci.

[30] Tan K L, Woon L L, Wong H K, Kang E T, Neoh K G (1993). Macromolecules.

[31] Liu Y, Ai K, Lu L (2014). Chem Rev.

[32] Kaspar T C, Droubay T, Chambers S A, Bagus P S (2010). J Phys Chem C.

[33] Firet N J, Blommaert M A, Burdyny T, Venugopal A, Bohra D, Longo A, Smith W A (2019). J Mater Chem A.

[34] Liu X-H, Cao Y-Y, Peng H-Y, Qian H-S, Yang X-Z, Zhang H-B (2014). CrystEngComm.

[35] Della Vecchia N F, Luchini A, Napolitano A, D’Errico G, Vitiello G, Szekely N, d’Ischia M, Paduano L (2014). Langmuir.

[36] Hong S, Na Y S, Choi S, Song I T, Kim W Y, Lee H (2012). Adv Funct Mater.

[37] Bernsmann F, Ball V, Addiego F, Ponche A, Michel M, de Almeida Gracio J J, Toniazzo V, Ruch D (2011). Langmuir.

[38] Kang X, Cai W, Zhang S, Cui S (2017). Polym Chem.

[39] Della Vecchia N F, Avolio R, Alfè M, Errico M E, Napolitano A, d'Ischia M (2013). Adv Funct Mater.

[40] Lee H, Dellatore S M, Miller W M, Messersmith P B (2007). Science.

[41] Lee B P, Dalsin J L, Messersmith P B (2002). Biomacromolecules.

[42] LaVoie M J, Ostaszewski B L, Weihofen A, Schlossmacher M G, Selkoe D (2005). Nat Med (Tokyo, Jpn).

[43] Wang W, Jiang Y, Wen S, Liu L, Zhang L (2012). J Colloid Interface Sci.

[44] Fei B, Qian B, Yang Z, Wang R, Liu W C, Mak C L, Xin J H (2008). Carbon.

[45] Liao Y, Wang Y, Feng X, Wang W, Xu F, Zhang L (2010). Mater Chem Phys.

[46] Liao Y, Cao B, Wang W-C, Zhang L, Wu D, Jin R (2009). Appl Surf Sci.

[47] Mekuye B, Ameen S, Akhtar M S, Jiménez-Suárez A (2023). The Impact of Size on the Optical Properties of Silver Nanoparticles Based on Dielectric Function. Nanotechnology and Nanomaterials Annual Volume 2024.

[48] Bellantone M, Williams H D, Hench L L (2002). Antimicrob Agents Chemother.

[49] Dai J, Bruening M L (2002). Nano Lett.

[50] Abdallah A S, Jande Y A C, Machunda R L (2019). Desalin Water Treat.

[51] Li Y, Sun Y, Zhang D, Xue Y, Wang J, Zhang N, Wang J, Zhang J, Zhao Y, Liu J-J (2024). Nano Res.

[52] Yang J, Chen Y, Jia X, Li Y, Wang S, Song H (2020). ACS Appl Mater Interfaces.

[53] Mansourpanah Y, Ghanbari A, Yazdani H, Mohammadi A G, Rahimpour A (2021). Desalination.

[54] Soroush A, Ma W, Silvino Y, Rahaman M S (2015). Environ Sci: Nano.

[55] Xu Y, Ma J, Han Y, Xu H, Wang Y, Qi D, Wang W (2020). Chem Eng J.

[56] Yoo C H, Jo Y, Shin J H, Jung S, Na J-G, Kang T, Lee J S (2022). Chem Eng J.

[57] Zhang M, Shi L, Du X, Li Z, Shi Y, An C, Li J, Wang C, Shi J (2022). Ind Crops Prod.

[58] Li S, Gao B, Wang Y, Jin B, Yue Q, Wang Z (2019). Desalination.

[59] Dong S, Hua H, Wu X, Mao X, Li N, Zhang X, Wang K, Yang S (2023). Environ Sci Pollut Res.

[60] Liu X, Foo L-X, Li Y, Lee J-Y, Cao B, Tang C Y (2016). Desalination.

[61] Dong C, Wang Z, Wu J, Wang Y, Wang J, Wang S (2017). Desalination.

[62] Widakdo J, Wu P-W, Austria H F M, Hung W-S, Yu P-J, Wang C-F, Hu C-C, Lee K-R, Lai J-Y (2022). Mater Today Chem.

[63] Liu C, Deng D, Xiao Z (2024). Desalination.

[64] Mozia S, Sienkiewicz P, Szymański K, Zgrzebnicki M, Darowna D, Czyżewski A, Morawski A W (2019). J Chem Technol Biotechnol.

[65] Zhu Z, He C, Sha J, Xiao K, Zhu L (2024). Sci Total Environ.

[66] Fan X, Peng Y, Li Y, Yang Y, You Z, Xu Y (2023). J Environ Chem Eng.

[67] Nguyen T P N, Yun E-T, Kim I-C, Kwon Y-N (2013). J Membr Sci.

