# Supplementary material for: Synthesis and antibacterial properties of nanosilver-modified cellulose triacetate membranes for seawater desalination
Source: Beilstein J Nanotechnol. 2025 Aug 19;16:1380–91. doi: 10.3762/bjnano.16.100 (PMC12415899; doi:10.3762/bjnano.16.100)
Supplement: File 1 — Additional experimental data. [file Beilstein_J_Nanotechnol-16-1380-s001.pdf]

BEILSTEIN JOURNAL OF NANOTECHNOLOGY

Supporting Information

for

Synthesis and antibacterial properties of nanosilver-modified cellulose triacetate membranes for seawater desalination

Lei Wang, Shizhe Li, Kexin Xu, Wenjun Li, Ying Li and Gang Liu

Beilstein J. Nanotechnol. **2025,** *16,* 1380–1391. doi:10.3762/bjnano.16.100

Additional experimental data

License and Terms: This is a supporting information file under the terms of the Creative Commons Attribution License (https://creativecommons.org/licenses/by/4.0). Please note that the reuse, redistribution and reproduction in particular requires that the author(s) and source are credited and that individual graphics may be subject to special legal provisions.
The license is subject to the *Beilstein Journal of Nanotechnology* terms and conditions: (https://www.beilstein-journals.org/bjnano/terms)

Calculation of PDA content in PCTA

According to our measurements, the dimensions of the PCTA membrane are 90 mm × 110 mm, with a thickness of approximately 0.022 mm. The weight of the CTA membrane is 0.223 g. To coat the CTA membrane, 8 mL of a dopamine solution with concentration 2 mg·mL^{-1} is used. So the total amount of PDA used for coating is:

$$Total\ \ PDA\ \ mass = 8\ \ \mathrm{mL} \times 2\ \ \mathrm{mg} \cdot \mathrm{mL}^{-1} = 16\ \ \mathrm{mg}$$

The mass ratio of PDA to CTA can be calculated as:

$$Mass\ \ ratio = \frac{16\ \ \mathrm{mg}}{0.233\ \ \mathrm{g}} \approx 0.068\ \ \mathrm{mg} \cdot \mathrm{mg}^{-1}$$

The surface area of the CTA membrane is:

$$Surface\ \ area = 90\ \ \mathrm{mm} \times 110\ \ \mathrm{mm} = 9900\ \ \mathrm{mm}^2$$

The amount of PDA per square millimeter is:

$$PDA\ \ per\ \ \mathrm{mm}^2 = \frac{16\ \ \mathrm{mg}}{9900\ \ \mathrm{mm}^2} \approx 0.0016\ \ \mathrm{mg} \cdot \mathrm{mm}^{-2}$$

Given the extremely low mass ratio (0.068 mg·mg^{-1}) and the low surface density (0.00016 mg mm^{-2}) of PDA relative to the CTA, the concentration of PDA is below the detection threshold of FTIR spectroscopy.

Determination of PDA and Ag content in Ag@PCTA

First, the Ag@PCTA membrane was prepared by coating an 8 mL solution of polydopamine (2 mg/mL) onto a CTA membrane with an area of 6800 mm^2 (85 mm × 80 mm) and a mass of 0.2034 g.

The mass ratio of PDA to CTA is calculated as:

$$Mass\ \ ratio\ \ of\ \ PDA\ \ to\ \ CTA = \frac{8\ \ \mathrm{mL} \times 2\ \ \mathrm{mg \cdot mL^{-1}}}{0.2034} = 0.079\ \ \mathrm{mg \cdot mg^{-1}}$$

Then, the Ag@PCTA membrane was prepared by coating a 5.1 mL solution of $Ag[NH_3]_2OH$ (0.002 mol·L^{-1}) onto the above PCTA membrane (area = 6800 mm^2, mass = 0.2034 g).

The mass ratio of silver to CTA is calculated as:

$$Mass\ \ ratio\ \ of\ \ Ag\ \ to\ \ CTA = \frac{5.1\ \ \mathrm{mL} \times 0.002\ \ \mathrm{mol \cdot L^{-1}} \times 107.87\ \ \mathrm{g \cdot mol^{-1}}}{0.2034}$$
$$\approx 0.0054\ \ \mathrm{mg \cdot mg^{-1}}$$

The PDA coating on PCTA and Ag@PCTA, while providing functional groups for further modification, does not introduce distinct IR peaks that are easily distinguishable from the CTA matrix. The low concentration of Ag nanoparticles in Ag@PCTA also results in minimal spectral changes compared to PCTA. The Ag nanoparticles are present in trace amounts, which may not significantly alter the overall FTIR spectrum.

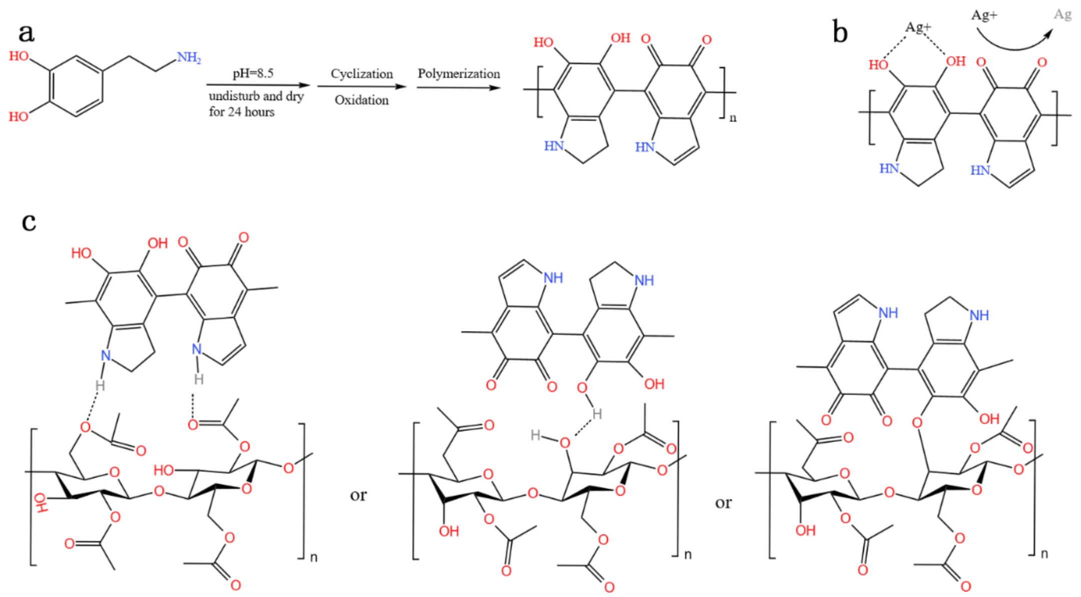

Figure S1: (a) The polymerization of dopamine under alkaline conditions, (b) the reduction and fixation of Ag by polydopamine, and (c) interactions between polydopamine and CTA including hydrogen bonding and ether substitution.

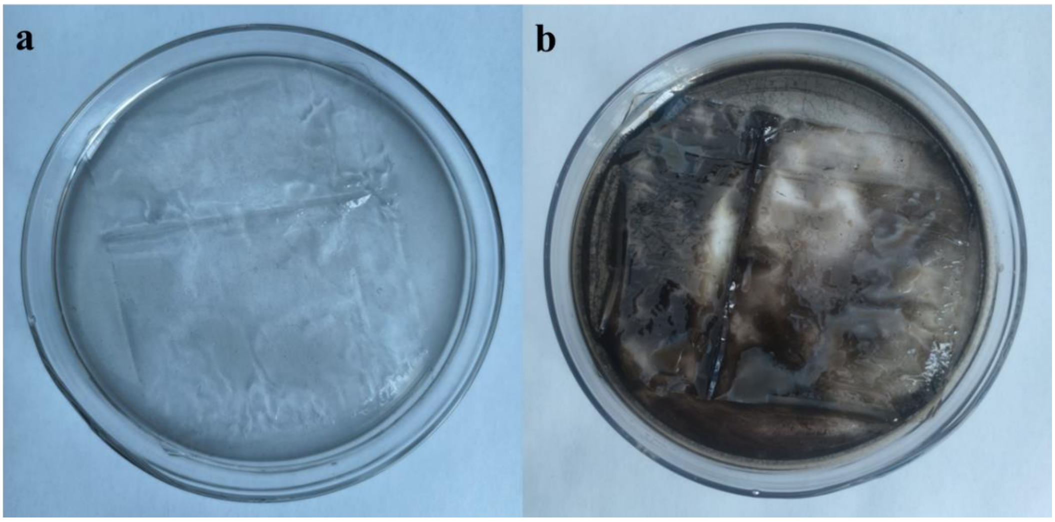

Figure S2: (a) Dopamine monomers are added into an alkaline solution in the initial stage, (b) polymerization to PDA after 24 h.

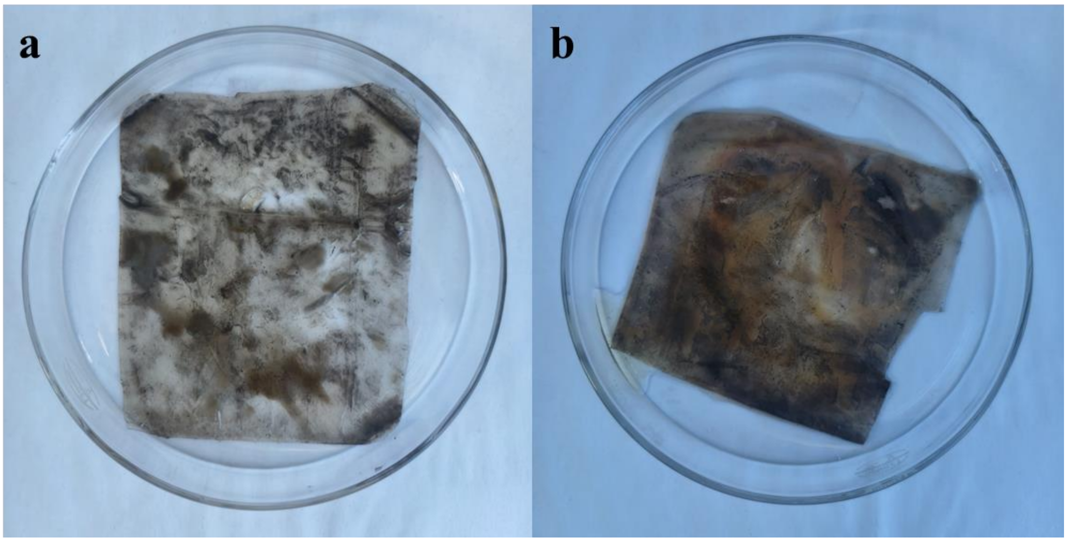

Figure S3: PCTA coated with $Ag[NH_3]_2OH$ (a) and not coated with $Ag[NH_3]_2OH$ solution (b).

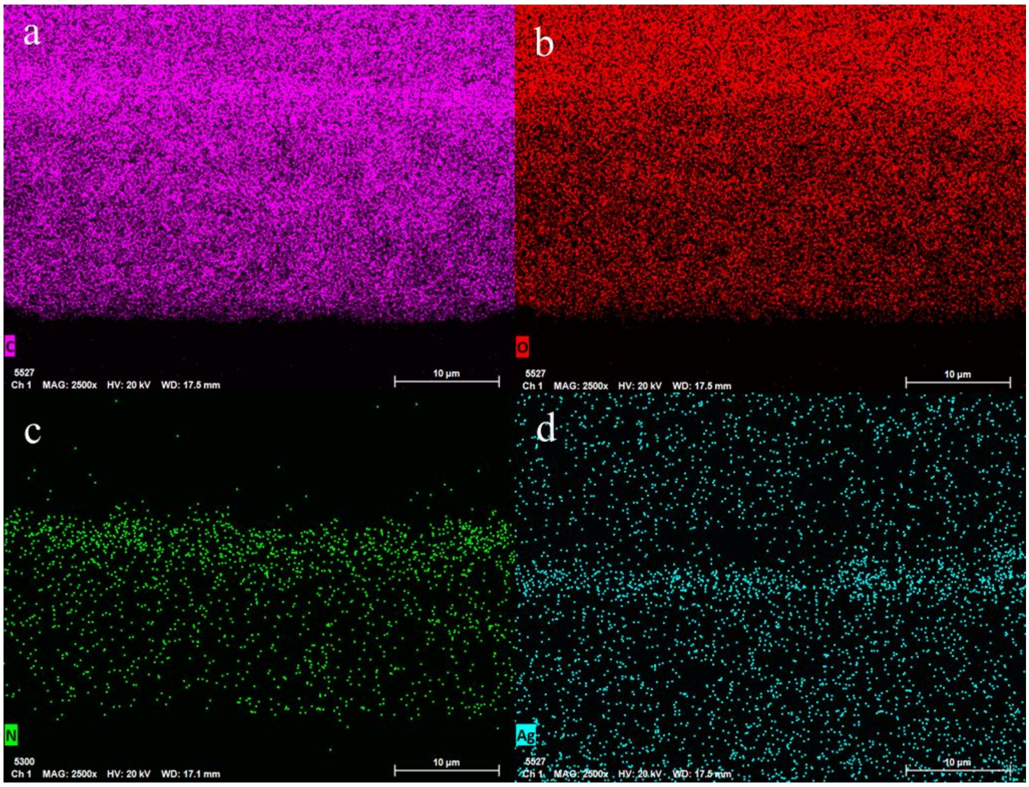

Figure S4: EDS elemental mappings show the distribution of elements in different materials: (a, b) C and O in CTA, respectively; (c) N in PCTA; (d) Ag in Ag@PCTA.

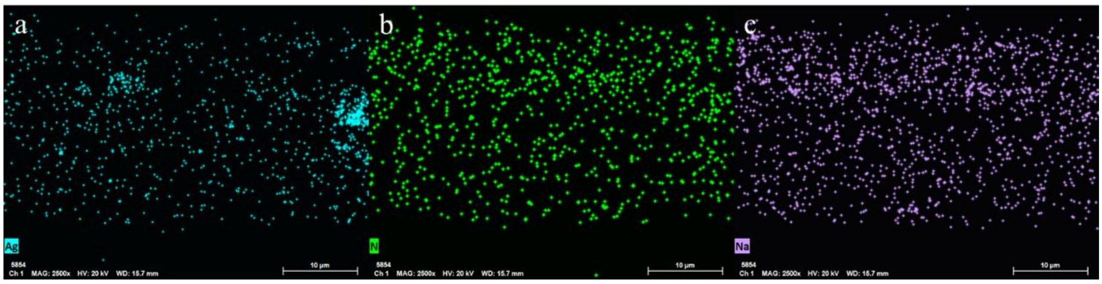

Figure S5: EDS of Ag, N, and Na within the layer of Ag@PCTA after desalination.
